# Increased risk of poor survival in ovarian cancer patients with high expression of SNAI2 and lymphovascular space invasion

**DOI:** 10.18632/oncotarget.14192

**Published:** 2016-12-25

**Authors:** Jun Li, Shufen Li, Ruifang Chen, Xin Lu

**Affiliations:** ^1^ Department of Gynecology, Obstetrics and Gynecology Hospital, Fudan University, Shanghai 200011, China; ^2^ Shanghai Key Laboratory of Female Reproductive Endocrine Related Diseases, Shanghai 200011, China; ^3^ State Key Laboratory of Medical Genomics and Shanghai Institute of Hematology, Ruijin Hospital, Shanghai Jiao Tong University School of Medicine, Shanghai 200025, China

**Keywords:** ovarian cancer, lymphovascular space invasion, SNAI2, prognosis, survival

## Abstract

This study is aimed to conduct a meta-analysis to evaluate the prognostic value of lymphovascular space invasion(LVSI) and to explore the potential association of SNAI1 and SNAI2 with LVSI in ovarian cancer. A systematic literature search in PubMed, ISI Web of Science, and Medline was conducted to identify relevant studies assessing the prognostic value of LVSI in ovarian cancer. The main outcomes analyzed were progression free survival/disease free survival and overall survival. TCGA database was used to explore the potential link of SNAI1 and SNAI2 with LVSI status. A total of 11 eligible studies enrolling 1817 patients were included for the meta-analysis. The overall analysis indicated that LVSI presence was associated with shorter duration of survival in ovarian cancer patients. Multivariate analysis indicated that both advanced stage and SNAI2 expression were associated with increased risk of LVSI presence. Survival analysis indicated that tumors with LVSI presence and high SNAI2 expression were significantly correlated with poorer survival when compared to tumors with both LVSI absence and low SNAI2 expression. In conclusion, LVSI presence was associated with worse clinical outcomes in ovarian cancer. Increased expression of SNAI2 and advanced stage were independent risk factors for LVSI presence. Our findings also emphasizes the potential of SNAI2 in promoting lymphovascular spread of ovarian cancer.

## INTRODUCTION

Ovarian cancer is the most fatal gynecologic malignancy and most of the patients are not diagnosed until an advanced stage [1]. During the past decade, researchers have been striving to identity potential prognostic predictors for ovarian cancer [2–5]. For example, debulking status has been associated with the clinical outcome [6, 7]. Namely, patients with optimal debulking surgery have a better survival than patients with suboptimal debulking surgery [6]. However, some optimally debulked cases present therapy resistance and developed recurrence, and subsequently have a worse outcome. At the other extreme, part of the suboptimally debulked cases exhibit a better therapy response, and thus have a better outcome. This fact indicated that other factors might play a critical role in determining survival. Identification of new factors associated with ovarian cancer prognosis will be helpful in stratifying patients who are likely to experience a disease progression to standard therapy and could benefit from alternative management [8].

Recently, lymphovascular space invasion (LVSI), defined as the detection of tumor cells inside the capillary lumens of lymphovascular system, has emerged as a new risk factor for ovarian cancer progression [9, 10]. The presence of LVSI was associated with a worse clinical outcome in ovarian cancer patients [9, 10]. However, some studies failed to uncover such an association [11, 12]. This discrepancy in results indicates that the prognostic value of LVSI in ovarian cancer remains controversial. Moreover, the underlying risk factors for LVSI presence in ovarian cancer remains largely elusive [9].

In present study, we first conducted a meta-analysis to establish the prognostic value of LVSI presence in ovarian cancer. Meanwhile, we try to explore the potential link between SNAI1, SNAI2, and LVSI status in ovarian cancer using data from The Cancer Genome Atlas (TCGA) database.

## RESULTS

### Characteristics of identified studies

**Figure 1:**
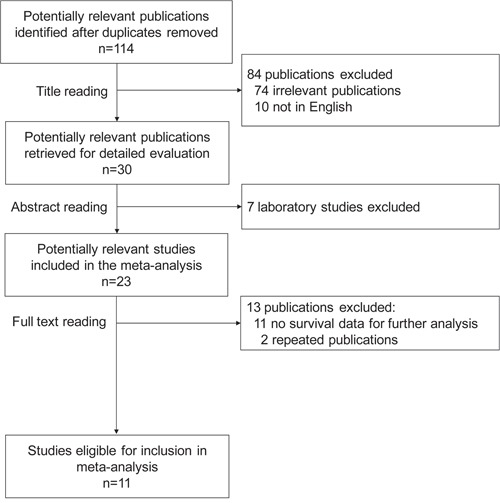
Flow chart of the search strategy used for selection of eligible studies

One hundred fourteen studies were identified by the primary computerized literature search. Of these, 91 publications were excluded because they were either irrelevant to the present study, written in non-English, or laboratory studies. Twenty-three records were further reviewed in detail. Thirteen publications were further excluded because of no survival data or repeated publications. The survival data on LVSI in the TCGA dataset was also included in our meta-analysis. Finally, 11 studies were identified as eligible for inclusion in our meta-analysis [6, 9–18] (Figure 1). The included 11 studies encompassed 1817 ovarian cancer patients. The main characteristics of the included studies are shown in Table 1.

**Table 1:** Characteristics of included studies

First author of study, y	Regions	No. of patients	Tumor stage (III-IV, %)	Grade	Histologic subtype	LVSI%	Cut-off value	Outcomes	HR estimation
Tomic 2003	Europe	80	III-IV, 66.3%	High, 50.0%	Not reported	68.80%	Presence	OS	Reported
Faleiro-Rodrigues 2004	Europe	104	III-IV, 63.5%	G3, 49.0%	Mixed(Serous, 53.8%)	29.80%	Presence	DFS, OS	Reported
Li 2009	Asia	78	III-IV, 56.4%	G2-G3, 67.9%	Mixed(Serous, 57.7%)	62.80%	Presence	DFS, OS	Reported
Chay 2013	Asia	107	III-IV, 14.0%	G2-G3, 36.6%	Mucinous	6.50%	Presence	PFS, OS	Reported
Matsuo 2014a	America	434	III-IV, 0.0%	G2-G3, 38.5%	Mixed(Serous, 13.1%)	17.50%	Presence	PFS, OS	Reported
Matsuo 2014b	America	121	III-IV, 95.0%	High, 95.0%	Serous	83.50%	Presence	PFS, OS	Reported
Chen 2015	Asia	492	III-IV, 68.7%	G2-G3, 91.0%	Mixed(Serous, 72.4%)	58.50%	Presence	PFS, OS	Reported
Masoumi-Moghaddam 2015	Asia	60	III-IV, 77.0%	G3, 77.0%	Mixed(Serous, 81%)	58.30%	Presence	DFS, OS	Reported
David 2016	Asia	68	III-IV, 30.9%	Not reported	Mixed(Serous, 23.5%)	19.10%	Presence	OS	Reported
Karan Krizanac 2016	Europe	81	III-IV, 87.7%	High, 86.4%	Serous	37.00%	Presence	DFS, OS	Reported
TCGA	Mixed	192	III-IV, 91.9%	G2-G3, 96.3%	Serous	70.80%	Presence	OS	Reported

### The effects of LVSI presence on survival in ovarian cancer

**Figure 2:**
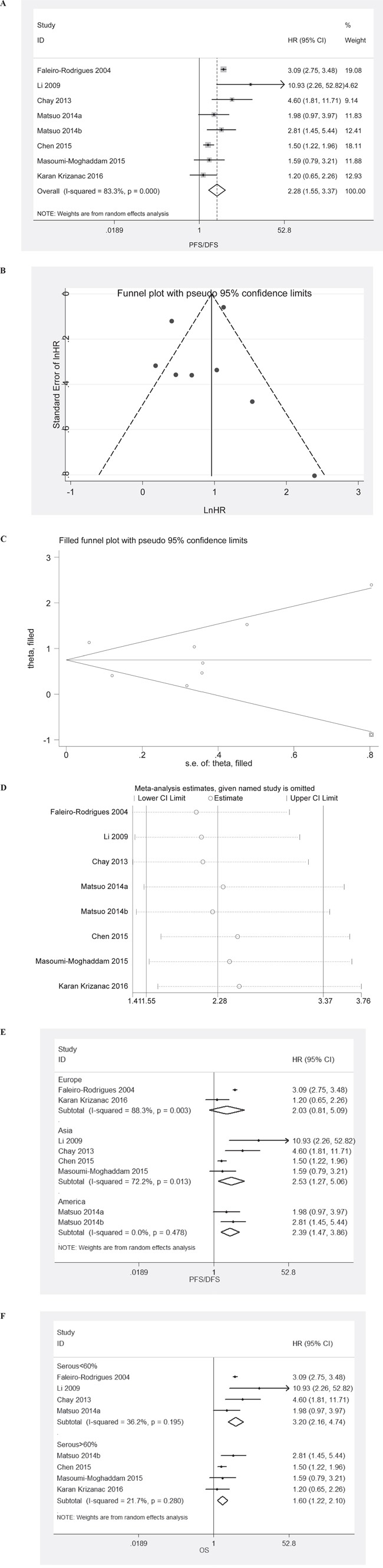
Meta-analysis of the HR for PFS/DFS for ovarian cancer patients depending on LVSI status **A**. PFS/DFS for ovarian cancer patients, random effects model; **B**. Assessment of publication bias by funnel plots; **C**. Identification of potential missing studies by the trim and fill analysis; **D**. Confirmation of the stability of the pooled results by one-way sensitivity analysis. **E**. Subgroup analyses stratified by region. **F**. Subgroup analyses stratified by histology.

HRs for PFS/DFS were available in 8 studies. The estimated pooled HR for all studies suggested a significantly increased risk of disease progression in patients with LVSI presence (Figure 2A; HR, 2.29; 95%CI, 1.55-3.37; P_HR_<0.001; random effects model). Funnel plot revealed that there was publication bias (Figure 2B). The trim-and-fill analysis revealed that one study might be missing. If this study were published, LVSI presence remained significantly correlated with disease progression (Figure 2C; HR, 2.12; 95%CI, 1.43–3.14; P_HR_<0.001; random effects model). One-way sensitivity analysis confirmed the stability of our results (Figure 2D). Subgroup analyses stratified by region and histology confirmed that LVSI presence was associated with increased risk of disease progression in all subgroups except the subgroup designated “Europe” (Figure 2E and 2F).

**Figure 3:**
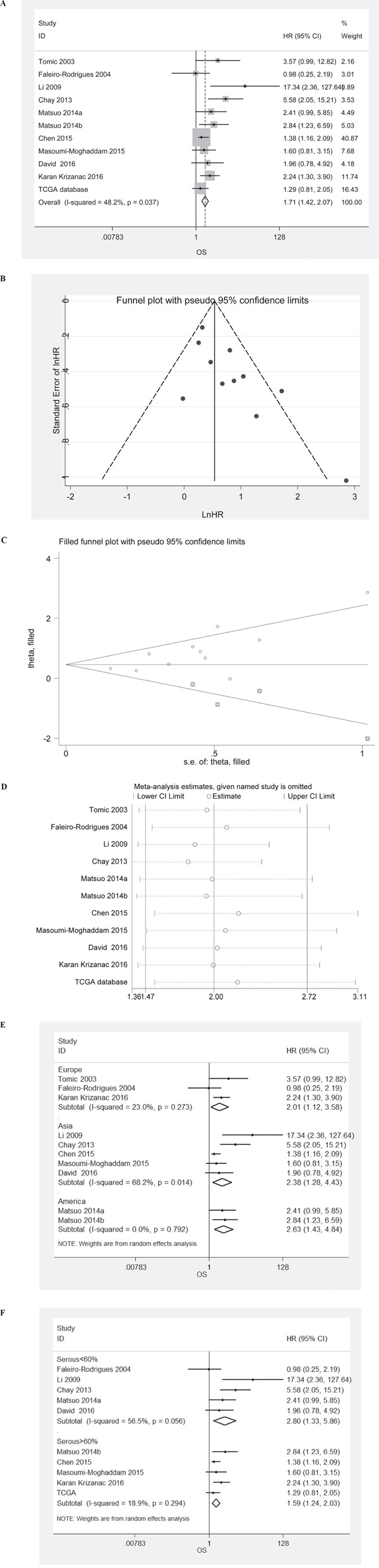
Meta-analysis of the HR for OS for ovarian cancer patients depending on LVSI status **A**. PFS/DFS for ovarian cancer patients, fixed effects model; **B**. Assessment of publication bias by funnel plots; **C**. Identification of potential missing studies by the trim and fill analysis; **D**. Confirmation of the stability of the pooled results by one-way sensitivity analysis. **E**. Subgroup analyses stratified by region. **F**. Subgroup analyses stratified by histology.

HRs for OS were available in 11 studies. The estimated pooled HR for all studies suggested a significantly increased risk of death in patients with LVSI presence (Figure 2B; HR, 1.71; 95%CI, 1.42-2.07; P_HR_<0.001; fixed effects model). Funnel plot revealed that there was publication bias (Figure 3B). The trim-and-fill analysis revealed that 4 studies might be missing. If these studies were published, LVSI presence remained significantly correlated with death of patients (Figure 3C; HR, 1.58; 95%CI, 1.13–2.22; P_HR_=0.008; random effects model). One-way sensitivity analysis confirmed the stability of our results (Figure 3D). Subgroup analyses stratified by region and histology confirmed that LVSI presence was associated with increased risk of death in all subgroups (Figure 3E & 3F).

**Figure 4:**
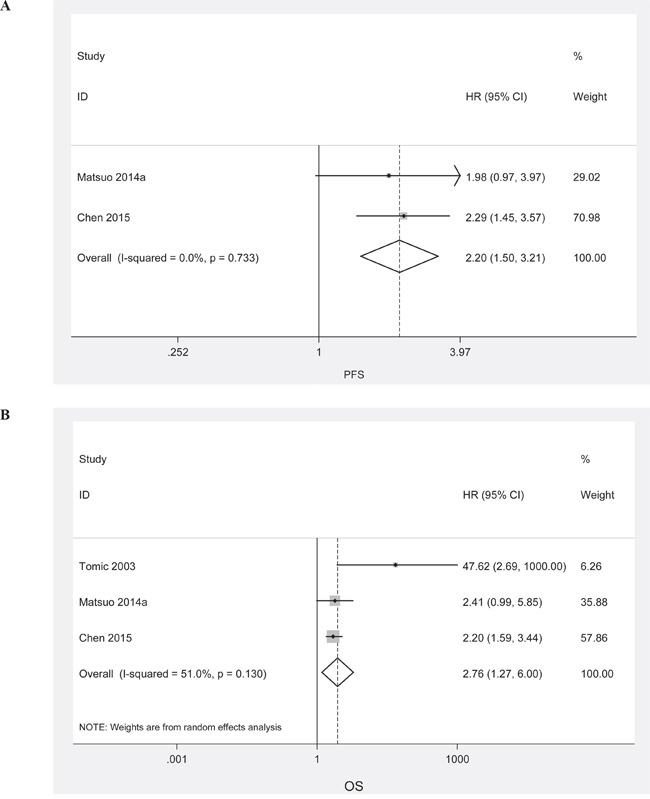
Meta-analysis of the HR for PFS and OS for early stage ovarian cancer patients depending on LVSI status **A**. PFS for early stage ovarian cancer patients, fixed effects model; **B**. OS for early stage ovarian cancer patients, random effects model.

Even patients at early stage had a relatively lower incidence of LVSI presence, LVSI presence was still associated with shorter duration of PFS (Figure 4A; HR, 2.20; 95%CI, 1.50-3.21; P_HR_<0.001; fixed effects model) and OS (Figure 4B; HR, 2.76; 95%CI, 1.27–6.00; P_HR_=0.011; random effects model).

### Identification of risk factors for LVSI presence in ovarian cancer patients from TCGA dataset

A total of 192 ovarian cancer patients with LVSI information from the TCGA dataset were included in this study. The clinical and pathological characteristics of the patients with or without LVSI are listed in Table 2. Advanced stage was significantly associated with increased risk of LVSI presence. Univariate analysis also revealed that the expression of SNAI1 and SNAI2 were all positively correlated with LVSI presence. In a multivariate model, advanced stage (OR, 4.44; 95%CI, 1.443-13.75; P =0.01) and SNAI2 expression (OR, 4.10; 95%CI, 1.78-9.47; P =0.001) remained significantly correlated with LVSI presence.

**Table 2:** Identification of risk factors for LVSI presence in ovarian cancer patients

Variables	Patients with LVSI		Patients without LVSI		Univariate analysis		Multivariate analysis	
	No. of patients	%	No. of patients	%	OR (95%CI)	P value	OR (95%CI)	P value
Age						0.334		/
<60	76	55.9%	27	48.2%	1		/	
≥60	60	44.1%	29	51.8%	0.74 (0.39-1.37)		/	
Histologic grade						0.604		/
G1	3	2.2%	2	3.6%	1		/	
G2-G3	131	96.3%	54	96.4%	1.62 (0.26-9.95)		/	
N/A	2	1.5%	0	0.0%	/		/	
Stage						0.006		0.01
I-II	11	8.1%	13	23.2%	1		1	
III-IV	125	91.9%	43	76.8%	3.44 (1.43-8.24)		4.44 (1.44-13.75)	
Cytoreduction						0.017		0.074
Optimal	84	61.8%	44	78.6%	1		1	
Sub-optimal	38	27.9%	7	12.5%	2.84 (1.17-6.89)		2.34 (0.92-5.97)	
N/A	14	10.3%	5	8.9%	/		/	
SNAI1						0.029		0.053
Low	52	38.2%	25	44.6%	1		1	
High	84	61.8%	31	55.4%	2.00 (1.07-3.76)		2.04 (0.99-4.21)	
SNAI2						<0.001		0.001
Low	68	50.0%	46	82.1%	1		1	
High	68	50.0%	10	17.9%	4.10 (2.15-9.86)		4.10 (1.78-9.47)	

**Figure 5:**
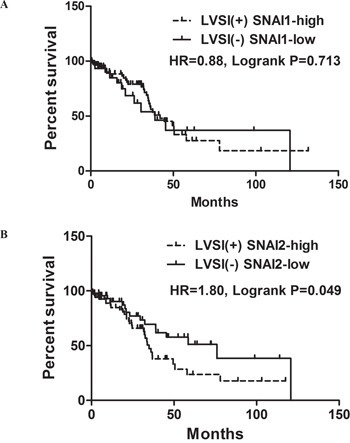
Survival curves with Kaplan-Meier method and Log-rank test for p-values **A**. OS for combination patters of LVSI and SNAI1; **B**. OS for combination patters of LVSI and SNAI2.

Next, survival analysis were conducted. When LVSI and SNAI1 are combined, the survival in patients with LVSI presence and high SNAI1 expression was not significantly inferior to that in patients with LVSI absence and low SNAI1 expression (Figure 5A; HR, 0.88; 95%CI, 0.44-1.76; P_HR_=0.713). However, patients with both LVSI presence and high SNAI2 expression were significantly associated with shorter OS when compared to patients with both LVSI absence and low SNAI2 expression (Figure 5B; HR, 1.80; 95%CI, 1.01-3.24; P_HR_=0.049).

## DISCUSSION

LVSI has been associated with worse prognosis of various cancers [19–21]. Consistently, our meta-analysis also indicated that LVSI presence was associated with shorter duration of PFS/DFS and OS in ovarian cancer. However, certain limitations should be considered when interpreting the pooled results. First, the present meta-analysis is based on the data from previously published retrospective studies, and the updated individual patient data were not integrated into the present analysis. Incorporation of updated individual data may further improve the accuracy and stability of the pooled findings. Second, significant heterogeneity existed in the present study. Variability in histology of ovarian cancer, patient population, and study design may result in the heterogeneity. Though our subgroup analyses supported the stability of our findings, multicenter prospective studies are strongly recommended to validate the prognostic value of LVSI in ovarian cancer. Third, publication bias is another concern. We tried to identify all relevant articles, but unavoidably, some articles could still be missing. Missing articles may contain negative results that could decrease the prognostic power of LVSI status. However, we performed the trim-and-fill analysis and found that even if these missing studies were included the association of LVSI presence and worse clinical outcome was still significant.

Although the prognostic value of LVSI has been established, the underlying risk factors responsible for LVSI presence in ovarian cancer are still largely elusive [9]. In present study, we showed that advanced stage was positively correlated with LVSI presence, which was consistent with the previous results [9]. Even the incidence of LVSI presence is lower in early stage cases than that in advanced stage cases, our meta-analysis also indicated that LVSI presence was still associated with poorer clinical outcome in early stage ovarian cancer patients.

SNAI2 is a key inducer of epithelial to mesenchymal transition (EMT). Previous results showed that SNAI2 increased the motile and invasive ability of ovarian cancer cells [22, 23]. This functional role of SNAI2 might facilitate the extravasation of ovarian cancer cells into surrounding tissues, for example, the capillary lumens of lymphovascular system. Interestingly, our data demonstrated that SNAI2 was an independent risk factor for LVSI presence. Previous results indicated that estrogen receptor expression was positively correlated with LVSI presence in ovarian cancer [9]. Additionally, SNAI2 was the downstream effector of estrogen receptor alpha pathway [24]. Whether there is a potential link between estrogen-signaling, SNAI2, and LVSI are not known and should be examined. Recently, multiple studies have indicated that circulating tumor cells (CTCs) status is an adverse prognostic factor for ovarian cancer [3, 25]. And it has also been demonstrated that EMT is conducive to CTCs generation and survival [26]. Whether CTCs is a mirror of LVSI are also not known and should be examined.

In conclusion, LVSI presence is associated with worse clinical outcomes. The aberrant expression of SNAI2 and advanced stage are independent risk factors for the LVSI presence in ovarian cancer. This study also emphasizes the potential and importance of SNAI2 in promoting lymphovascular spread of ovarian cancer.

## MATERIALS AND METHODS

### Search strategy

A literature search (last search updated to Oct.20th 2016) in Pubmed, ISI Web of Science, and Medline for studies evaluating the prognostic significance of LVSI in ovarian cancer was conducted using the following keywords: (“lymphovascular invasion” OR “lymphatic invasion” OR “vascular invasion” OR “LVSI”) AND (“ovarian cancer” OR “ovarian tumor” OR “ovarian carcinoma” OR “ovarian neoplasms”). Additionally, references lists of retrieved articles were checked for any possible eligible studies. The results were limited to peer-reviewed, English language reports.

### Eligibility criteria

The studies were considered eligible if they reported survival data in ovarian cancer patients stratified by LVSI status and provided sufficient data for determining an estimate of hazard ratio (HR) and a 95% confidence interval (CI). All articles were scrutinized to avoid inclusion of duplicate data. When the patient populations overlapped between studies, only the most recent or most complete publication was included to avoid duplications.

### Data extraction and outcomes

The data extracted for this meta-analysis included the author's names, year of publication, number of patients analyzed, tumor stage, grade, histology, and survival data stratified by LVSI status.

### TCGA dataset

We downloaded the level 3 Affymetrix HG-U133A gene expression data from 192 serous ovarian cancer patients with LVSI information in TCGA dataset to analyze the association of LVSI status with the clinicopathological features, and to determine the risk factors associated with LVSI presence.

### Statistical analysis

HR of each study was extracted directly from the original report. The potential heterogeneity between studies was assessed by the Cochran's Q-test and expressed by the I^2^ index. The pooled HR for survival was calculated by fixed-effects model when the I^2^≤50%. Otherwise, random-effects model was used. Publication bias was assessed by the funnel plot. The impact of publication bias on the pooled HR was evaluated with the trim-and-fill analysis. Moreover, one-way sensitivity analyses and subgroup analyses were performed to assess the stability of the results. When the number of included studies was less than three, one-way sensitivity analysis was not performed. All statistical tests of the meta-analysis were conducted with STATA version 11.0.

Risk factors related to LVSI presence was assessed with binary logistic regression test. Multivariate analysis with logistic regression test was conducted to identify independent risk factors for LVSI presence. The optimal cutoff for SNAI1 and SNAI2 was determined by Youden's index. The cutoff with the biggest Youden's index was chosen as the optimal cutoff. Statistical analysis was conducted using SPSS version 16.0.
